# Quantitative analysis of piroxicam using temperature-controlled ionic liquid dispersive liquid phase microextraction followed by stopped-flow injection spectrofluorimetry

**DOI:** 10.1186/2008-2231-21-63

**Published:** 2013-07-29

**Authors:** Mohsen Zeeb, Parisa Tayebi Jamil, Ali Berenjian, Mohammad Reza Ganjali, Mohamad Reza Talei Bavil Olyai

**Affiliations:** 1 Department of Applied Chemistry, Faculty of science, Islamic Azad University, South Tehran Branch, Tehran, Iran; 2 Center of Excellence in Electrochemistry, Faculty of Chemistry, University of Tehran, Tehran, Iran

**Keywords:** 1-Hexyl-3-methylimidazolium hexafluorophosphate, Stopped-flow injection spectrofluorimetry, Pharmaceutical formulations, Biological samples

## Abstract

**Background:**

Piroxicam (PXM) belongs to the wide class of non-steroidal anti-inflammatory drugs (NSAIDs). PXM has been widely applied in the treatment of rheumatoid arthritis, gonarthrosis, osteoarthritis, backaches, neuralgia, mialgia. In the presented work, a green and benign sample pretreatment method called temperature-controlled ionic liquid dispersive liquid phase microextraction (TCIL-DLPME) was followed with stopped-flow injection spectrofluorimetry (SFIS) for quantitation of PXM in pharmaceutical formulations and biological samples.

**Methods:**

Temperature-controlled ionic liquid dispersive liquid phase microextraction (TCIL-DLPME) was applied as an environmentally friendly sample enrichment method to extract and isolate PXM prior to quantitation. Dispersion of 1-hexyl-3-methylimidazolium hexafluorophosphate ([Hmim][PF_6_]) ionic liquid (IL) through the sample aqueous solution was performed by applying a relatively high temperature. PXM was extracted into the extractor, and after phase separation, PXM in the final solution was determined by stopped-flow injection spectrofluorimetry (SFIS).

**Results and Major Conclusion:**

Different factors affecting the designed method such as IL amount, diluting agent, pH and temperature were investigated in details and optimized. The method provided a linear dynamic range of 0.2-150 μg l^-1^, a limit of detection (LOD) of 0.046 μg l^-1^ and a relative standard deviation (RSD) of 3.1%. Furthermore, in order to demonstrate the analytical applicability of the recommended method, it was applied for quantitation of PXM in real samples.

## Background

Piroxicam (PXM, 4-hydroxy-2-methyl-N-(pyridine-2-yl)-2H-1, 2-benzo-thiazine-3-carboxamide-1,2-dioxide) belongs to the wide class of non-steroidal anti-inflammatory and analgesic drugs (NSAIDs) [1]. This drug has been widely used to treat podagrous and rheumatoid arthritis, gonarthrosis, osteoarthritis, backaches, neuralgia, mialgia, and other diseases accompanied by the pain syndrome or an inflammatory process [2,3]. To our knowledge, until now, some analytical techniques such as high performance liquid chromatography (HPLC) [4], capillary electrophoresis [5], liquid chromatography-mass spectrometry (LC/MS) [6], spectrometry [7], electrochemistry [8] have been reported for the quantitative analysis of PXM. The data obtained in these works reveal that the sensitivity and selectivity is not acceptable which is due to low amount of analyte in real sample and matrix impact. Thus, development and application of practical and benign sample pretreatment procedures prior to quantitation are important tasks of chemists. Combination of sample enrichment procedures with inexpensive, selective and sensitive determination tools such as spectrofluorimetry makes it possible to determine trace levels of analytes, provide better selectivity and extremely reduce the cost of analysis.

Previous studies reveal that ionic liquids (ILs) are suitable materials in sample enrichment procedures due to their special properties [9]. One of the practical advantages of ILs application in microextraction methods is the removal of toxic extraction materials. The most popular extraction techniques based on ILs are ionic liquid-based dispersive liquid-liquid microextraction (IL-DLLME) [10-13], cold-induced aggregation microextraction (CIAME) [14] and temperature-controlled ionic liquid dispersive liquid phase microextraction (TCIL-DLPME) [15].

In TCIL-DLPME procedure, dispersion of IL through the sample aqueous solution is occurred by applying a relatively high temperature. This phenomenon increases the chance of analyte extraction into extractor phase. By cooling the solution and centrifugation, it is possible to collect the IL-phase and transfer it to analytical tool for subsequent analysis. Based on the data obtained in our previous works, the out put of ionic liquid-sample enrichment methods meaningfully depends on variations in the values of ionic strength [16-19]. It is obvious that this factor can affect on the solubility of extractor. In order to obtained stable results, a common ion of extractor solvent (PF_6_^-^) was dissolved in the studied solution. Using this way, the volume of the extractor was not affected by changes occurred in the value of ionic strength.

In this study, spectrofluorimetric method was utilized for quantitation owning to some advantages including good selectivity and sensitivity, low cost of analysis and high response speed. To our knowledge, for the first time, TCIL-DLPME was combined with stopped-flow injection spectrofluorimetry (SFIS) for trace determination of PXM. The factors influencing the proposed method were studied in details and optimized. Finally, In order to demonstrate the analytical advantage of TCIL-DLPME-SFIS, It was utilized for quantitation of PXM in pharmaceutical and biological samples.

## Material and methods

### Instrumentation

Fluorescence signals were recorded using FP-6200 spectrofluorimeter (JASCO Corporation, Tokyo, Japan). Xenon discharge lamp, peristaltic sipper unit (model SHP-292), and micro-cell (path length of 3 mm and volume of 15 μL) were used as the accessories. The PC-based Windows® Spectra Manager™ software was applied for recording and processing of the analytical signals. A centrifuge was purchased from Hettich (Tuttlingen, Germany) and used for accelerating the phase separation. The pH-meter model 692 (Herisau, Switzerland) supplied with a glass-combined electrode was used for pH measuring.

### Reagents and materials

Analytical-reagent grade of chemicals was used in all experiments. All aqueous solutions were prepared using ultra pure water. Piroxicam hydrochloride was obtained from Alhavi Pharmaceutical Company (Tehran, Iran). 1-Hexyl-3-methylimidazolium hexafluorophosphate [Hmim][PF_6_], acetone, acetonitrile, methanol, ethanol, NH_3_, HCl, NaOH and sodium hexafluorophosphate (NaPF_6_) were purchased from Merck (Darmstadt, Germany). A stock solution of 200 mg ml^-1^ of NaPF_6_ was obtained by dissolving required amount of NaPF_6_ in water. Piroxicam has a low solubility in water (0.00004 M). Hence, a stock solution of piroxicam (1000 mg l^-1^) was obtained by dissolving 10 mg of pure drug in 10 ml of 5 M NH_3_. The obtained stock solution was stored at 5°C in the dark until use. Working solutions of lower concentrations were prepared daily from the above stock solution as required. Piroxicam capsules and tablets were obtained from a local pharmacy.

### Microextraction procedure

In the presented microextraction procedure, aliquots of 10.0 ml sample solution (pH = 3) containing PXM in the concentration range of 0.2-150 μg l^-1^ were placed into a screw-glass test tube with conic bottom. Then, 55 mg of [Hmim][PF_6_] IL and 0.9 ml of NaPF_6_ (200 mg ml^-1^) was added. After shaking, the conical tube was heated in a water bath with the temperature controlled at 40 °C for 5 min. Under this condition, the extractor was dissolved completely and the PXM was effectively extracted into the IL phase. After this step, the resulting solution was placed in ice-water bath and cooled for 7 min. After this process, a turbid condition was formed due to the decrease of the solubility of the extractor. The obtained solution was centrifuged for 5 min at 4000 rpm. The aqueous phase was removed using a proper syringe. For conditioning the extractor prior to quantitation by SFIS, the residue in the vessel was diluted to 250 μL by adding required amount of ethanol. Finally, the diluted IL-phase in the vessel was transferred to the spectrofluorimeter by the peristaltic sipper unit.

### Stopped-flow injection spectrofluorimetry

**Figure 1:**
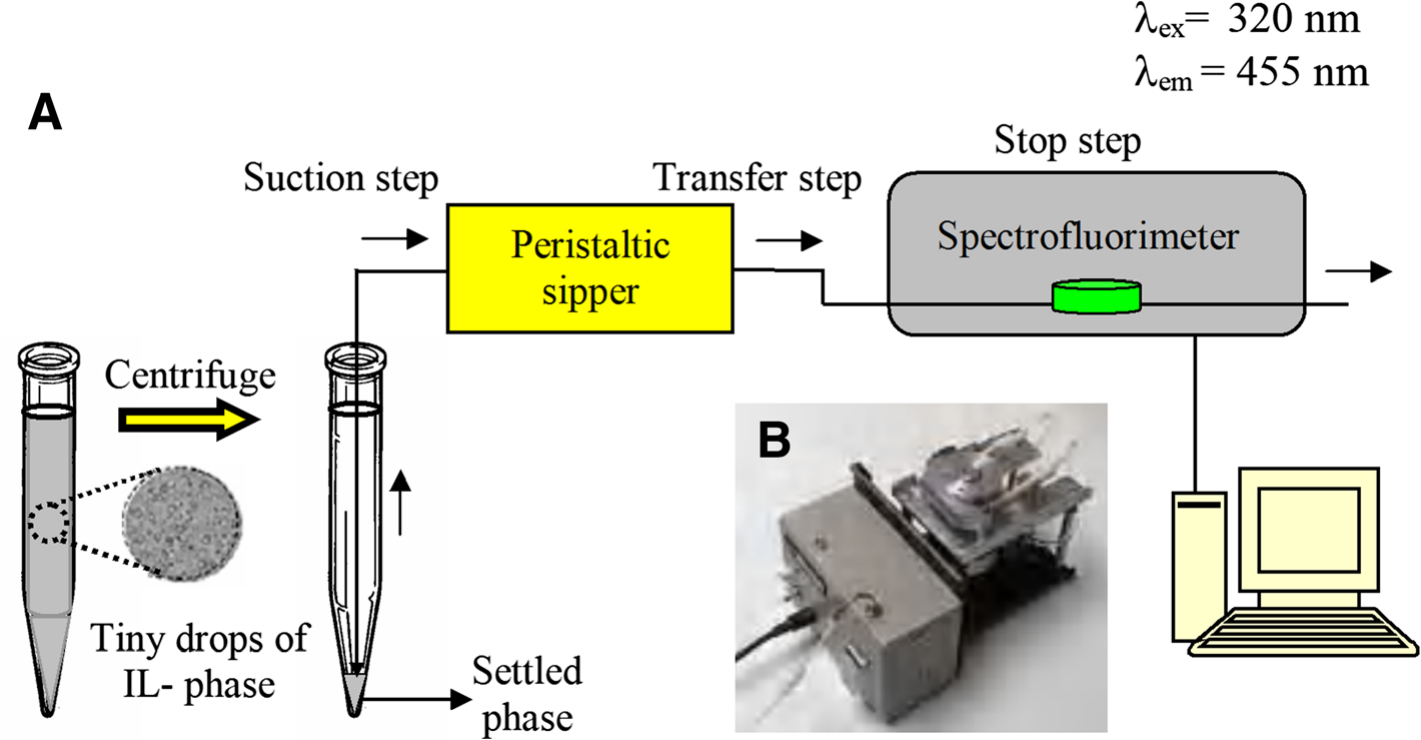
**(A) Schematic diagram of SFIS (B) Structure of peristaltic sipper unit.** Utilized experimental conditions: λ_ex_ 320 ± 5 nm; λ_em_ 455 ± 5 nm; suction time 0.4 s; transferring time 1 s; delay time 2 s.

In the presented work, fluorescence signals were recorded using stopped-flow injection technique. In order to apply stopped-flow injection mode, a peristaltic sipper unit supplied with a 15 μL quartz micro-cell was utilized to increase the speed of measurement and significantly reduce the required volume of the extractor. In order to control the enriched phase uptake, the rotation time of the peristaltic pump was changed. For transferring the extraction solvent to the spectrofluorimeter, the tube of the peristaltic pump unit was placed into the sample vessel, and suction step was started for 0.4 s. After this process, IL-phase was introduced to the 15 μL micro-cell. This step was applied for 1 s. In order to apply a delay time, the rotation of peristaltic pump was stopped. The stopped time was applied for 2 s, in order to obtain a stable experimental condition prior to quantitation. Fluorescence signal was recorded at 455 ± 5 nm. The excitation wavelength was fixed at 320 ± 5 nm. Schematic diagram of stopped-flow injection spectrofluorimetry applied in the present study is shown in Figure 1.

### Preparation of pharmaceutical formulations and biological samples

In order to obtain analyzable pharmaceutical solution, six piroxicam capsules or powdered tablets were entirely mixed, and afterwards a proper amount of powder containing 10 mg of PXM was dissolved in 5 M NH_3_. To prepare a clear solution, the resulting sample was filtered into a 100 ml volumetric flask by Whatman No. 42 filter paper, and made up to the mark using ultra pure water. Prior to quantitation, a proper dilution was performed, in order to ensure the concentration of the pharmaceutical solution was in the dynamic range.

In order to obtain analyzable human plasma samples, 1.0 ml of this real sample were spiked with PXM and deproteinized by addition of 5 ml acetonitrile. After this process, the resulting biological real sample was centrifuged at 4000 rpm for 15 min, and 2.0 ml of the clear upper pahse was diluted to 100 ml. Aliquot of 10 ml of this sample was utilized for each test. 10 ml of human urine samples were transferred into centrifuge tubes. Urine samples were centrifuged for 4 min at 4000 rpm. Afterwards, aliquots of 2 ml from clear upper phase were transferred into new centrifuge tubes, spiked with different concentrations of PXM and diluted to 20 ml. In order to determine the trace levels of PXM, aliquot of 10 ml of this solution was subjected to TCIL-DLPME-SFIS.

## Results and discussion

In the recommend method, an efficient sample pretreatment method called temperature-controlled ionic liquid dispersive liquid phase microextraction (TCIL-DLPME) was followed by stopped-flow injection spectrofluorimetry (SFIS) for preconcentration and trace level determination of PXM in real samples. To achieve a proper efficiency and stability, different variables affecting the recommended method were studied and optimized. Pre-concentration factor (PF) was evaluated according to the following equation:

$$\mathrm{PF}=\frac{\mathrm{Csed}}{C0}$$

In this equation, C_sed_ and C_0_ show the concentration of PXM in the enriched phase and initial concentration of PXM in the aqueous phase, respectively. C_sed_, for extractor solvents and diluting agents, was measured using the calibration graph obtained from direct injection of PXM in enriched phase.

### Spectrofluorimetric calibration curve and spectral characteristics

**Figure 2:**
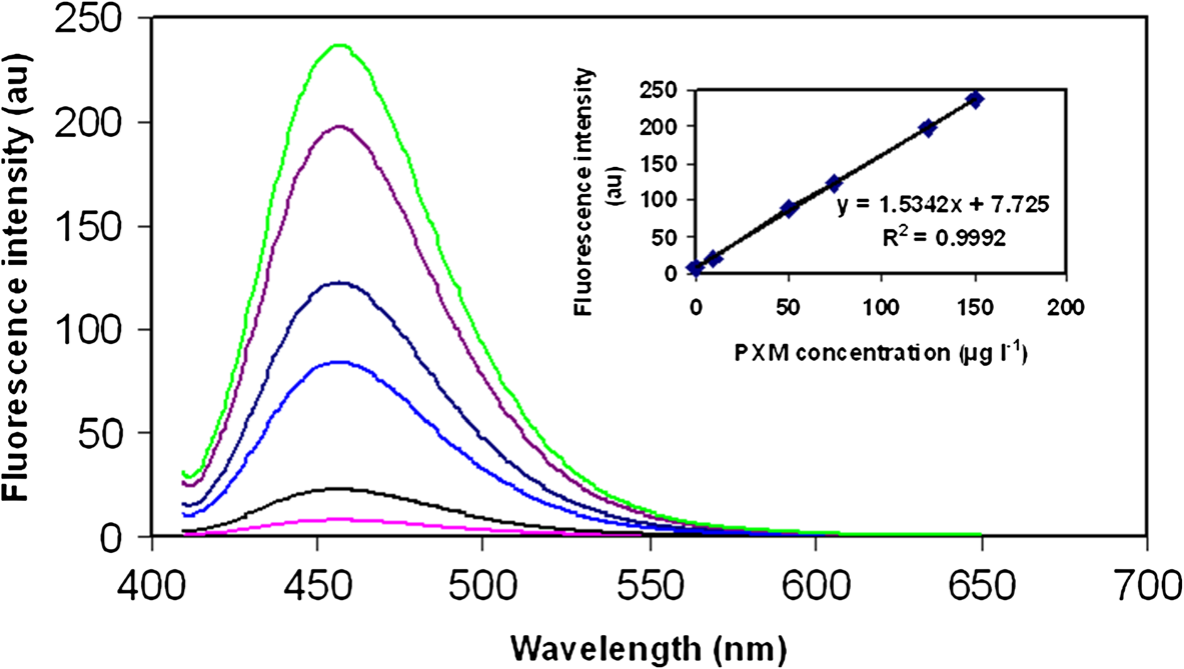
**Fluorescence spectra obtained for 6 standard solutions of PXM with different concentrations (0.2, 10, 50, 75, 125, 150 μg l**^**-1**^**).** Utilized experimental conditions: Sample volume 10 ml; [Hmim][PF_6_] 55 mg; NaPF_6_ 180 mg; pH 3; temperature 40°C; centrifugation time 5 min. λ_ex_ 320 ± 5 nm; λ_em_ 455 ± 5 nm. Inset: Calibration curve and corresponding equation in the linear range of analytical signals.

Since PXM has a cyclic conjugated structure, this compound shows considerable fluorescence signal. The emission spectra of 6 standard solutions of PXM with different concentrations in the range of 0.2-150 μg l^-1^ were recorded using TCIL-DLPME-SFIS (see Figure 2). Fluorescence signal was recorded at 455 ± 5 nm. The excitation wavelength was fixed at 320 ± 5 nm. To achieve stable, reproducible and accurate data, the reagent blank must have no measurable impact on the fluorescence signal of PXM. Hence, the sample enrichment procedure was applied for reagent blank using the mentioned excitation and emission wavelengths. The data obtained in this test shown no significant impact. Hence, the mentioned wavelengths were selected for the rest of the work.

### Selection of IL

Following consideration can ease the selection of IL: (a) the density of extractor must be higher than aqueous phase, (b) extractor must be liquid through the experiments, (c) IL must show proper hydrophobic manner and (d) IL must be certainly inexpensive. ILs containing (CF_3_SO_2_)_2_N^−^ are relatively expensive and those containing PF_6_^-^ are relatively inexpensive. According to the mentioned points, [Hmim][PF_6_] was selected as a microextraction solvent in all experiments.

### Type of diluting agent

Kind of diluting agent is one the important factors in TCIL-DLPME. Since the density of ionic liquid is relatively high, this extractor must be diluted prior to transfer and quantitation. In the present test, some organic diluting agent involving methanol, ethanol, acetone and acetonitrile were investigated. Because of the better performance of ethanol and its better safety, this diluting material was utilized in all the tests.

### Influence of IL amount

**Figure 3:**
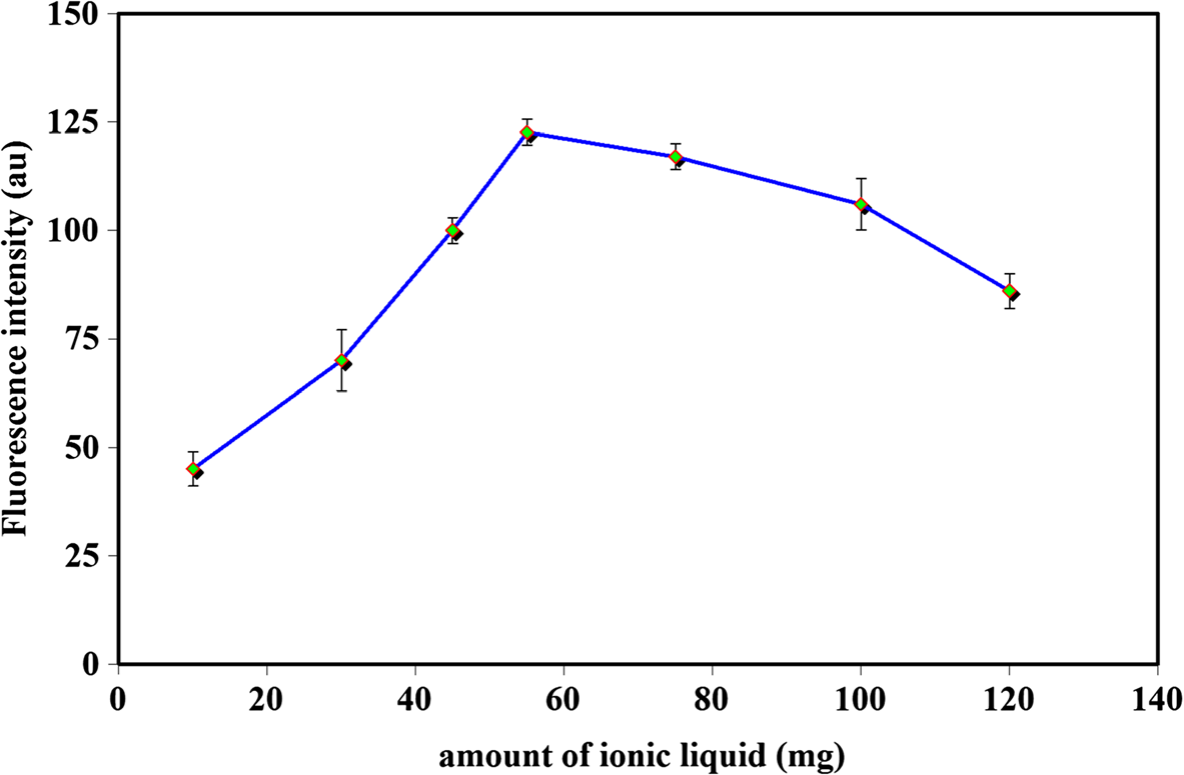
**Effetc of IL amount.** Utilized experimental conditions: Sample volume 10 ml; PXM concentration 75 μg l^-1^; NaPF_6_ 180 mg; pH 3; temperature 40°C; centrifugation time 5 min. λ_ex_ 320 ± 5 nm; λ_em_ 455 ± 5 nm.

In this investigation, the influence of [Hmim][PF_6_] amount on the TCIL-DLPME and subsequent analytical signals was tested. The influence of this factor was tested within the range of 10–120 mg (Figure 3). Stable data were achieved at 55 mg of IL. When a larger amount of extractor to be used, the volume of the enriched phase increases and the signal intensity decreases. As a result, 55 mg of extractor was selected as an optimum value and used in the following experiments.

### Influence of common ion amount and ionic strength

**Figure 4:**
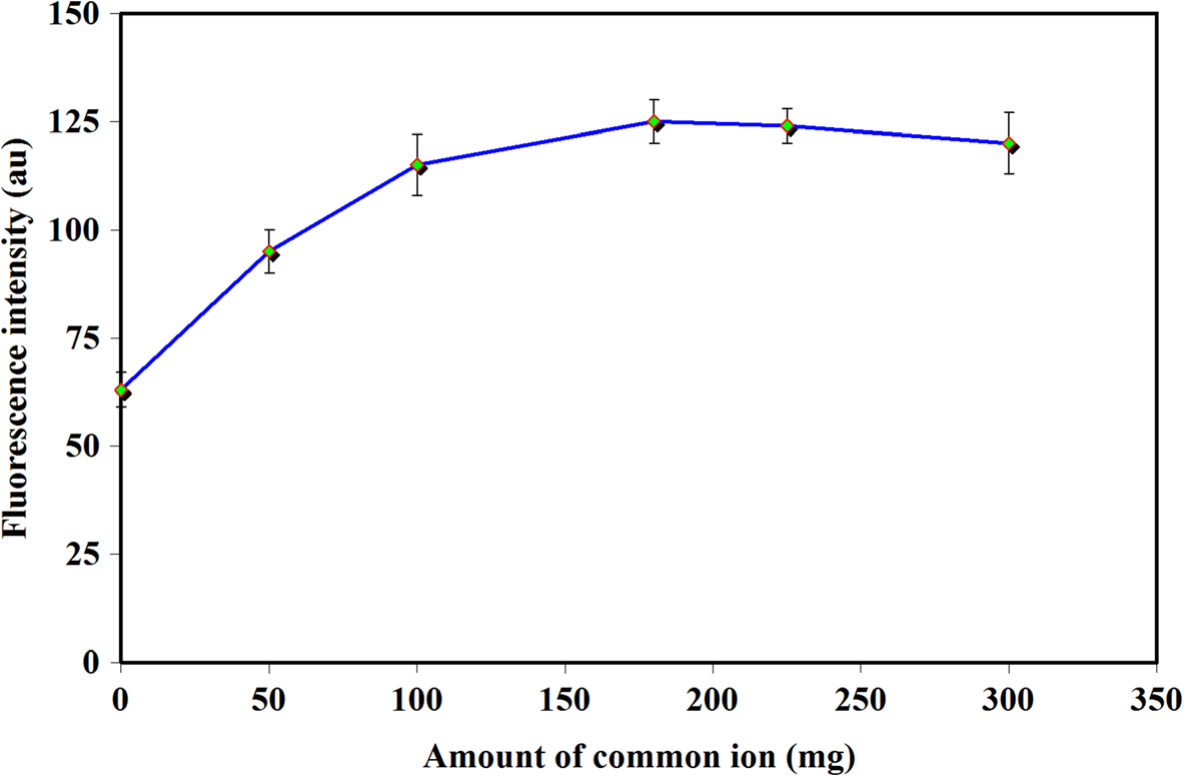
**Effect of common ion.** Utilized experimental conditions: Sample volume 10 ml; PXM concentration 75 μg l^-1^; [Hmim][PF_6_] 55 mg; pH 3; temperature 40°C; centrifugation time 5 min. λ_ex_ 320 ± 5 nm; λ_em_ 455 ± 5 nm.

Based on the results obtained in our previous works, dissolving a common ion of extractor can provide more stable dada. As it was described, this act can decrease the solubility of the extraction phase and provide better sensitivity. For this evaluation, NaPF_6_ was applied in all tests a common ion source. The impact of this factor was tested in the range of 0–300 mg. Figure 4 show the obtained results in this experiment. A better stability was achieved at 180 mg. Therefore, this amount was applied for the rest of the work.

Based on the results obtained in our previous studies, in traditional sample enrichment methods based on ILs, the volume of remaining enriched phase depends on the value of the ionic strength. As it was explained above, in order to overcome this phenomenon, a common ion of extractor phase was dissolved in the sample under study. NaNO_3_ was utilized as an electrolyte to test the impact of this factor. This factor was carefully evaluated over the range of 0–35% (w/v) and no measurable impact was observed in this range.

### Influence of pH

**Figure 5:**
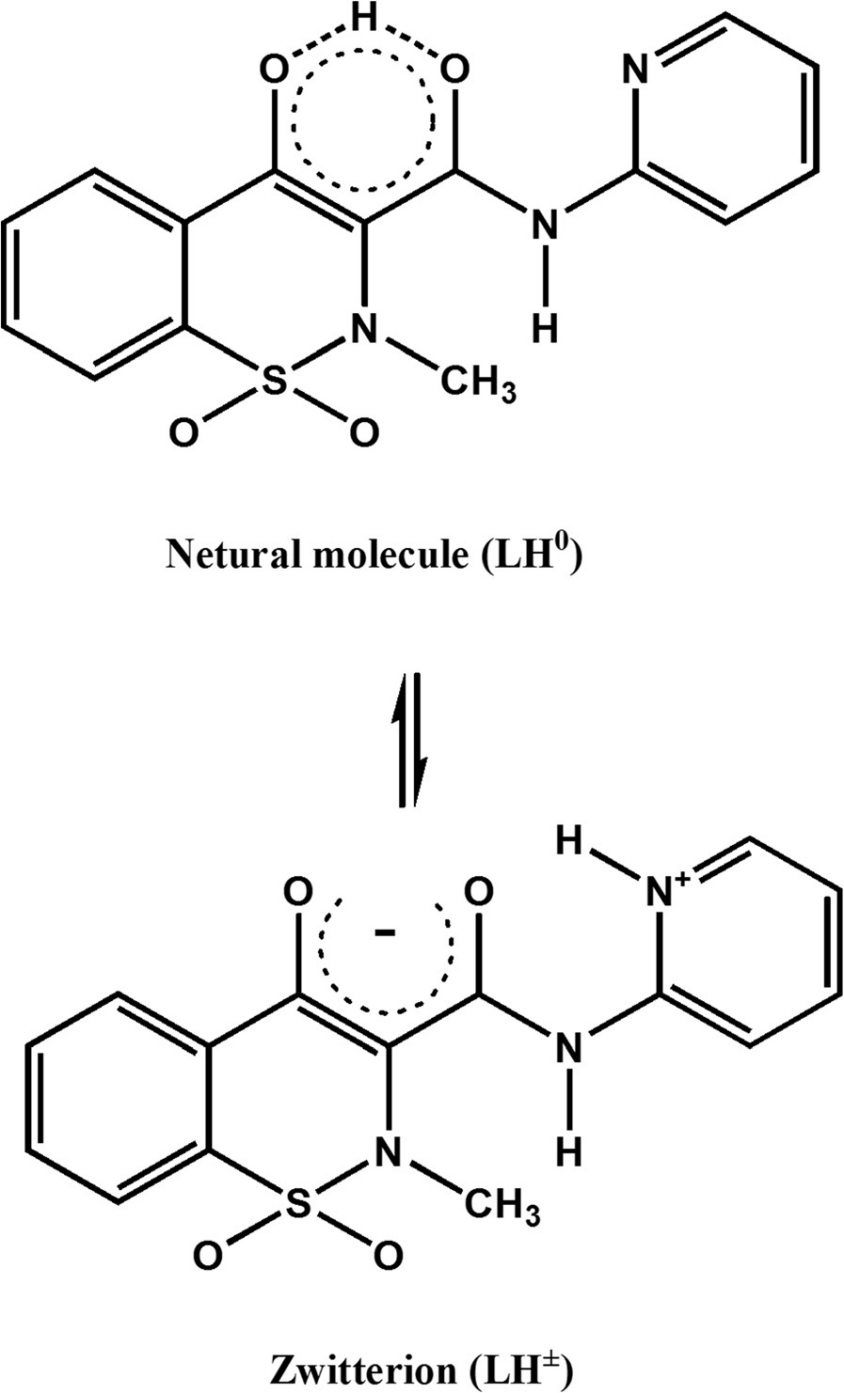
**Equilibrium of PXM between the neutral molecule (LH**^**0**^**) and the zwitterion (LH**^**±**^**).**

**Figure 6:**
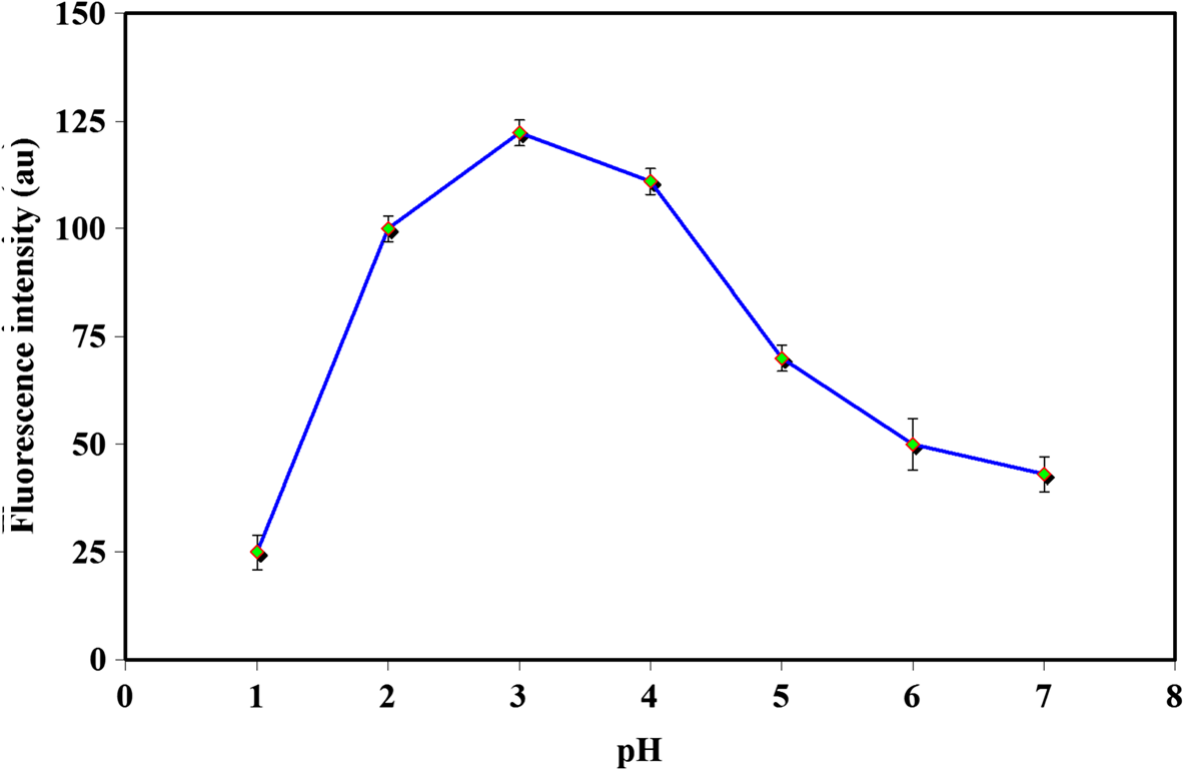
**Effect of pH.** Utilized experimental conditions: Sample volume 10 ml; PXM concentration 75 μg l^-1^; [Hmim][PF_6_] 55 mg; NaPF_6_ 180 mg; temperature 40°C; centrifugation time 5 min. λ_ex_ 320 ± 5 nm; λ_em_ 455 ± 5 nm.

The pH of the sample solution plays a critical role in the extraction of ionizable organic molecules such as PXM. The influence of this factor on the microextraction of PXM was evaluated within the range of 1–8 using HCl and NaOH. The best data is obtained at pH values where the uncharged condition of the compound of interest is prevalent. The reported acidic constants for PXM are pK_a1_ = 1.81 and pK_a2_ = 5.12 [20]. Figure 5 shows a possible equilibrium between the neutral molecule (LH^0^) and the zwitterion (LH^±^). The variation of analytical signals versus pH revealed that a better extraction was obtained at pH 3 (Figure 6). Thus, pH 3 was used in the following study.

### Influence of temperature

Complete dispersion of IL-phase in the sample solution is occurred by applying a relatively high temperature. The obtained data revealed that the solubility of extractor at temperature above 30°C significantly increased. A series experiments were performed in order to optimize the temperature in the range of 30–70°C. A better stability and sensitivity was obtained at 40°C, therefore this temperature was used an optimum value. In the next step, sample solutions were cooled in the temperature range of 0-25°C. By applying a low temperature, the analytical sensitivities improve which is due to the solubility decrease of extractor phase at low temperatures. Hence, a temperature of 0°C was utilized through the rest of the work.

### Influence of centrifuge conditions

In this experiment, the impact of this factor on the sensitivity and reproducibility was tested in the range of 1000–6000 rpm. In order to settle the extractor phase, 3500 was suitable. Hence, 4000 rpm was chosen as the optimum value. After this study, the impact of centrifugation time on the sensitivity and stability of signals was tested. After 4 min, no measurable change was observed. As a result, 5 min was selected as the centrifugation time. Table 1 shows the tested and selected experimental conditions.

**Table 1:** Evaluated and optimized experimental conditions of TCIL-DLPME-SFIS

**Microextraction factors**	**Evaluated range**	**Optimized value**
Amount of [Hmim][PF_6_] ionic liquid (mg)	10-120	55
Amount of common ion (NaPF_6_) (mg)	0-300	180
pH	1-8	3
Temperature (°C)	30-70	40
Centrifugation rate (rpm)	1000-6000	4000
Centrifugation time (min)	> 2	5
**Instrumental factors**	**Evaluated range**	**Optimized value**
Suction time (s)	0.1-3	0.4
Transferring time (s)	0.1-3	1
Delay time (s)	0.1-4	2
Excitation wavelength (nm)	200-400	320 ± 5
Emission wavelength (nm)	410-700	455 ± 5
Excitation and emission slit widths (nm)	5-20	10

### Selectivity of the method

In the present study, in order to demonstrate the selectivity of the method, the impact of some possible interfering substances such as Na^+^, Ca^2+^, Zn^2+^, Mg^2+^, Cl^-^, PO_4_^3-^, SO_4_^2-^, citric acid, starch, glucose, lactose, sucrose, ascorbic acid, uric acid, oxalic acid and lactic acid on the determination of PXM at 75 μg L^-1^ was tested. 100-fold the mentioned substances have no observable impact on the fluorescence responses (fluorescence response change below 5%).

### Method evaluation

In order to obtain calibration graph and linear dynamic range, different concentrations of PXM standard solutions were subjected to TCIL-DLPME-SFIS. The designed method provided a linear dynamic range of 0.2-150 μg l^-1^ PXM. Analytical performance of the combined methodology is summarized in Table 2. The limit of detection (LOD) of TCIL-DLPME-SFIS was defined using the following equation:

$$\text{LOD}={\text{ks}}_{\text{bl}}/m.$$

**Table 2:** Analytical performance of the proposed methodology

**Analytical parameter**	**Performance**
Dynamic range (μg L^-1^)	0.2-150
Correlation coefficient (R^2^)	0.9992
LOD^a^ (μg L^-1^)	0.046
RSD^b^ (%) (n = 4 ) (C_PXM_ = 75 μg L^-1^)	3.1
PF^c^ (C_PXM_ = 75 μg L^-1^)	31.2
Sample volume (mL)	10
Temperature (°C)	40

^a^ Limit of detection.

^b^ Relative standard deviation.

^c^ Preconcentration factor.

In the mentioned equation, the value of K is 3, s_bl_ shows the standard deviation of the blank signals and m shows the calibration slope. By this way, the LOD found was 0.046 μg l^-1^. In order to define the repeatability of the designed system, four 75 μg l^-1^ standard solutions of PXM were analyzed and by this way the relative standard deviation (RSD) was 3.1%.

### Analysis of PXM in real samples

The analytical applicability of the designed TCIL-DLPME-SFIS was tested by the quantitation of PXM in the spiked human urine and spiked human plasma. These data are summarized in Table 3. These tests revealed that the mean recoveries of PXM in studied real biological samples were in the range of 96–103% and 95.2-104% for plasma and urine samples, respectively. The obtained data demonstrated the acceptable accuracy and precision of the designed methodology. In the next experiments, the proposed technique was utilized for the quantitation of PXM in pharmaceutical formulations including tablets and capsules. The obtained data are shown in Table 3. The recent data show the validity of the designed TCIL-DLPME-SFIS for the determination of PXM in pharmaceutical formulations.

**Table 3:** **Determination of PXM in real samples using TCIL-DLPME-SFIS**^**a**^

**Sample**	**Added (μg l**^**-1**^**)**	**Found (μg l**^**-1**^**)**	**Recovery (%)**	**RSD (%)**
Human plasma	0.0	-	-	-
	5.0	4.8	96	4.0
	75.0	73.2	97.6	3.3
	150.0	148.3	98.9	3.9
Human urine	0.0	-	-	-
	5.0	5.2	104	3.0
	75.0	72.8	97.1	3.1
	150.0	144.0	96	4.3
Prioxicam capsule	0.0	9.6	-	-
	5.0	14.3	97.9	3.9
	75.0	80.6	95.3	3.2
	150.0	147.1	98.1	3.0
Prioxicam tablet	0.0	9.7	-	-
	5	14.0	95.2	4.2
	75.0	80.9	95.5	3.0
	150.0	146.3	97.5	4.7

^a^ The illustrated results correspond to the average of three independent measurements.

## Conclusion

In this study, a benign and simple sample enrichment method called temperature- controlled ionic liquid dispersive liquid phase microextraction (TCIL-DLPME) was followed by stopped-flow injection spectrofluorimetry (SFIS) for quantitation of piroxicam in pharmaceutical and biological samples. Ionic liquid was used an extractor instead of toxic solvents, in order to protect the environment against harmful material and provide a better safety for chemists during the experiments. Traditional sample enrichment methods based on the application of ionic liquid suffer from some limitations such as the dependence of extraction efficiency on ionic strength values. To remove the latter problem, the microextraction procedure was assisted by a common ion of extractor. The application of stopped-flow injection mode makes it possible to increase the speed of quantitative measurements, decrease the required enriched phase and provide a better reproducibility and automation. Fluorimetry was applied as a determination technique because of some advantages including good selectivity and sensitivity, low cost of analysis and high response speed. For quality control of PXM, the present methodology is an efficient, benign, simple and inexpensive analytical tool.

## Competing interests

The authors declare that they have no competing interests.

## Authors’ contributions

All authors contributed equally. All authors read and approved the final manuscript.
